# L1 cell adhesion molecule high expression is associated with poor prognosis in surgically resected brain metastases from lung adenocarcinoma

**DOI:** 10.1016/j.clinsp.2022.100040

**Published:** 2022-05-04

**Authors:** Jia-Wei Wang, Song-Quan Wang, Zhuo-Yi Wu, Qi Liu, Qing Yuan, Hong-Qing Cai, Jing-Hai Wan

**Affiliations:** Department of Neurosurgery, National Cancer Center/National Clinical Research Center for Cancer/Cancer Hospital, Chinese Academy of Medical Sciences and Peking Union Medical College, PR China

**Keywords:** L1 cell adhesion molecule, Prognostic factor, Brain metastasis from lung cancer, Neurosurgical resection

## Abstract

**Objectives:**

Accurate prognosis assessment across the heterogeneous population of brain metastases is very important, which may facilitate clinical decision-making and appropriate stratification of future clinical trials. Previous studies have shown the L1 Cell Adhesion Molecule (L1CAM) is potentially involved in human malignancies of multiple different samples and unfavorable survival. However, no data of L1CAM are available for the brain metastases from lung adenocarcinoma, especially for the one with neurosurgical resection.

**Method:**

The authors investigated the L1CAM expression in cranial metastatic lesions for patients with brain metastases from lung adenocarcinoma after neurosurgical resection using tissue microarrays that were obtained from the Department of Neurosurgery at the Cancer Hospital of the Chinese Academy of Medical Sciences. Furthermore, the relationship between L1CAM expression and clinic-pathological parameters, including overall survival time, was analyzed to assess the prognostic value of L1CAM.

**Results:**

L1CAM high expression was found in 62.30% of brain metastases from lung adenocarcinoma and significantly correlated with brain metastasis number (p = 0.028) and Lung-molGPA score (p = 0.042). Moreover, L1CAM expression was an independent predictor of survival for brain metastases after neurosurgical resection in a multivariate analysis. Patients with L1CAM high expression had unfavorable overall survival time (p = 0.016). In addition, the multivariate analysis also showed age and extracranial transfer were also the independent prognostic factors for this type of patient with brain metastases.

**Conclusions:**

A subset of brain metastases from lung adenocarcinoma aberrantly expresses L1CAM. L1CAM is a novel independent prognostic factor for brain metastasis from lung adenocarcinoma after neurosurgical resection.

## Highlights

• Brain metastasis aberrantly expresses L1CAM.
• L1CAM high expression is associated with poor prognosis in brain metastases.

## Introduction

As the leading cause of cancer-related mortality in China and worldwide, lung cancer accounts for > 20% of all cancer-related deaths.^1^ It is reported that most lung cancer-associated deaths result from metastasis, especially brain metastasis. 20%‒60% of patients with lung cancer may develop brain metastasis during the course of their disease. Moreover, with the recent advance of molecularly targeted therapies and immunotherapies, survival from lung cancer continues to improve and patients are thus at greater risk for developing brain metastasis. In addition, the majority of brain metastasis results from lung cancer, predominantly from the adenocarcinoma subtype. Actually, brain metastasis from lung cancer has become a seriously social health burden around the world.

Unfortunately, although the well-known deleterious effects of brain metastasis from lung cancer have long been recognized, medical and surgical interventions remain limited, and advances in treatment have been modest.^2^^,^^3^ To date, a growing number of molecularly targeted therapies and immunotherapies for lung cancer are now available and/or are under active investigation. However, several factors, such as barriers in the central nervous system, different molecular profiles, and the response of metastatic cells in the CNS to targeted drugs, restrict the efficacy of these systemic therapies in brain metastasis. Thus, local therapies, such as neurosurgical resection, radiotherapy, including Stereotactic Radiosurgery (SRS), and Whole Brain Radiation Therapy (WBRT), remain the mainstay of treatment for the brain metastasis from lung cancer.^3^ Extensive efforts have been focused on predicting outcomes for this extremely heterogeneous population of patients who develop brain metastases.

As a member of the immunoglobulin supergene family, L1CAM (L1 cell adhesion molecule) is initially found to be involved in CNS development, such as neuronal differentiation and migration.^4^ In the past few years, a host of research have been carried out around the world to discuss the expression and function of L1CAM in human malignancies of different patient samples.^5^, ^6^, ^7^, ^8^, ^9^, ^10^, ^11^, ^12^, ^13^ It has been demonstrated as a predictive factor of poor prognosis in Non-Small Cell Lung Cancer (NSCLC), cervical cancer, endometrial cancer, gastric cancer, glioma, renal cell cancer, ovarian carcinoma, melanoma, colon cancer, and pancreatic cancer. In addition, previous studies have also indicated the involvement of L1CAM in the tumor cell invasion, metastasis, and chemoresistance. Currently, no data on L1CAM are available for brain metastasis samples from lung cancer.

The present study aimed to investigate the relationship between L1CAM expression in cranial metastatic lesions and clinicopathological parameters in the population of patients with brain metastases from lung adenocarcinoma after neurosurgical resection. Further, the authors tested the hypothesis that L1CAM expression was relevant for survival time in this subtype of patients.

## Materials and methods

### General materials

Cranial tumor tissues of neurosurgical resected brain metastases from lung adenocarcinoma between 2014 and 2019 were obtained from a tissue bank in the Department of Neurosurgery at the National Cancer Center (NCC), Cancer Hospital of the Chinese Academy of Medical Sciences and used for the construction of tissue microarray.^8^ Histotypes such as small cell, large cell, squamous, neuroendocrine or sarcomatoid carcinoma, and other types of metastatic tumors were not included. Data concerning the clinical/pathological parameters of each patient were recorded, including age/sex of patients, smoking history, Karnofsky Performance Status (KPS) scores, locations and numbers of brain metastases, extracranial transfer, pathology subtype, gene alterations, treatment information, Overall Survival (OS) time and the Updated Graded Prognostic Assessment for Lung Cancer Using Molecular Markers (Lung-molGPA).^14^ OS time was defined as the interval between the date of diagnosis of brain metastases and the date of death from all causes or the date of the last follow-up for survivors. Sixty-one brain metastases from lung adenocarcinoma with complete data were enrolled in the final analysis. This research was performed in accordance with the World Medical Association Declaration of Helsinki and was approved by the Ethics Committee of the Cancer Hospital, Chinese Academy of Medical Sciences (Reference number NCC2021C-516/22/052-3253).

### TMA construction

TMAs were constructed as described previously with a minor modification in the study's laboratory.^15^, ^16^, ^17^ Briefly, formalin-fixed, paraffin-embedded tissue blocks and corresponding histological H&E-stained slides were used for TMA sampling. Two experienced investigators reviewed the slides together to determine and label representative areas of tumor tissue. 1.0 mm diameter cylinders of tumor tissue in triplicate were punched from selected tumor areas of individual tissue blocks to control for potential tumor heterogeneity and were re-embedded in recipient paraffin blocks at defined positions using a tissue arraying instrument (Beecher Instruments, Silver Spring, MD). The TMA block contained 61 brain metastasis samples from lung adenocarcinoma. Subsequently, multiple 4 μm sections were sliced from the TMA block and mounted on microscope slides. One section from the tissue array block was stained with H&E to confirm that the punches contained tumor tissue.

### Immunohistochemistry (IHC) of L1CAM and assessment of IHC

IHC of the TMA sections was performed as previously described standard technique.^17^ The primary antibody L1CAM (mouse monoclonal antibody, Sigma Aldrich, St. Louis, MO, USA) was used and tested on a multi-tissue TMA for appropriate dilutions (1:1000). As a negative control, alternative TMA sections were incubated without a primary antibody. IHC Images were taken on an Olympus microscope with CCD camera using the analysis software (Olympus, Munster, Germany).

**Fig. 1:**
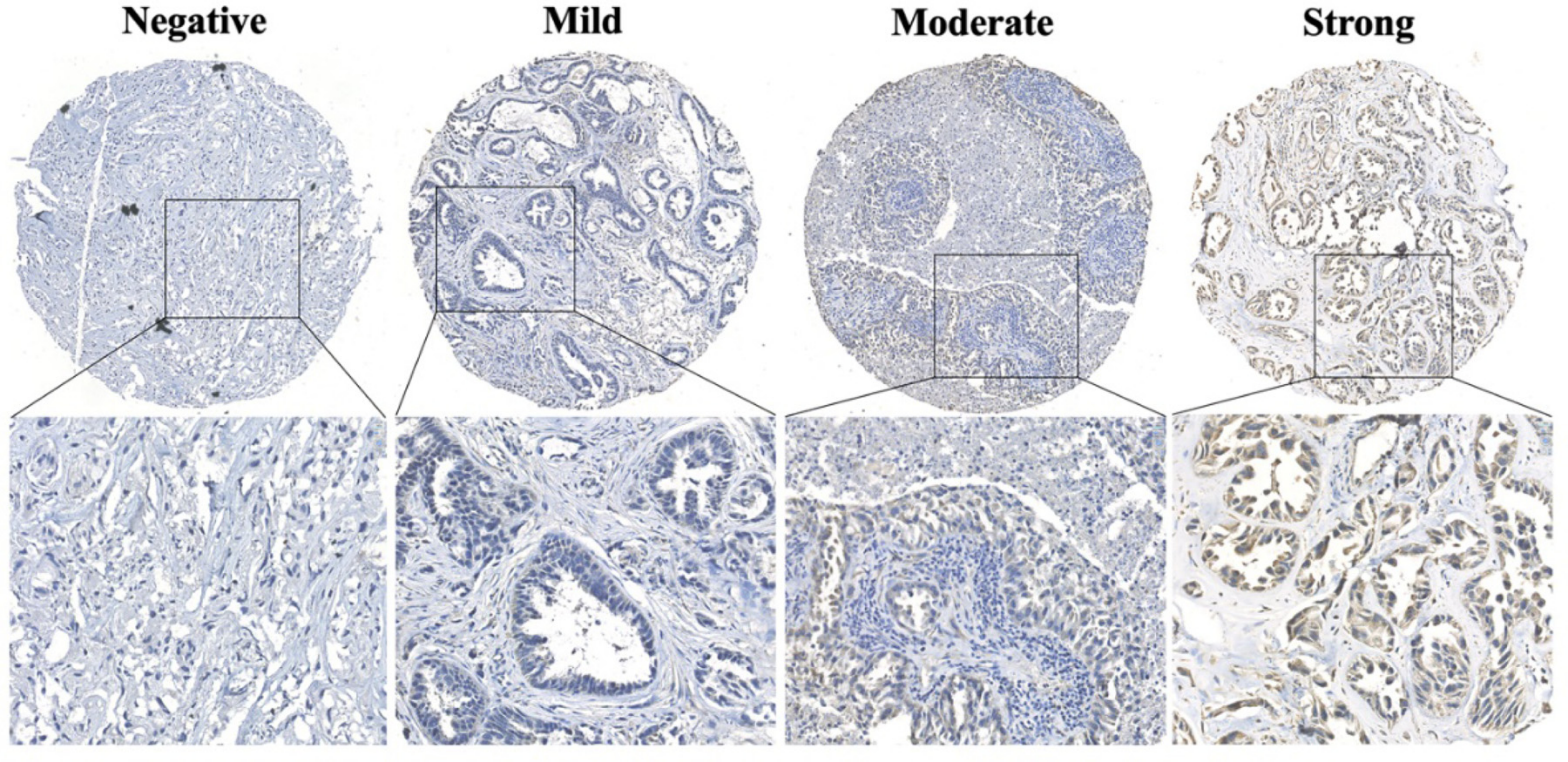
Typical L1CAM stained images on TMA section of brain metastases from lung adenocarcinoma, including negative, mild, moderate, and strong staining. L1CAM, L1 Cell Adhesion Molecule; TMA, Tissue Microarray.

Immunohistochemical evaluation of L1CAM was independently performed by two experienced investigators who were blinded to patients' characteristics and outcomes, with discrepancies resolved by consensus under a microscope for multi-viewing. The immunoreactivity of L1CAM was semi-quantitatively scored, applying an adjusted Allred scoring system. In brief, the 0‒3 point staining intensity score was used as described in Fig. 1: 0, no staining; 1, weak staining; 2, moderate staining; and 3, strong staining. Furthermore, the proportion score of positively stained tumor cells in slides was recorded as a percentage  × 100. Finally, the total value for immunoreactivity of L1CAM was 0‒300 by staining intensity × proportion scores. The scores for tumors with multiple sections were averaged, which represented the final score of each specimen. X-tile software (Version 3.6.1, Yale University, USA) was used to ascertain the optimal cut-off point of L1CAM (high expression vs low expression) for subsequent survival analysis.

### Statistical analysis

All statistical analyses were performed using SPSS Statistics software version 26 (SPSS, Inc., Chicago, IL, USA). The Chi χ2 test was used to assess the relationship between L1CAM expression and clinic-pathological parameters. OS curves were plotted according to the Kaplan-Meier method, with the log-rank test applied for comparison. Multiple Cox proportional hazard model was used to predict independent prognostic factors that significantly impacted patient survival; p-value of less than 0.05 was considered statistically significant.

## Results

### L1CAM expression in brain metastases from lung adenocarcinoma

An overview of L1CAM expression and clinicopathological parameters is shown in Table 1. In addition, Fig. 1 demonstrated typical L1CAM stained images on the TMA section, including negative, mild, moderate, and strong staining. Six-one patients (Male: thirty-five, Female: twenty-six) with brain metastases from lung adenocarcinoma were enrolled in the final analysis, with the age range from 29 to 85 years old. Among these patients, L1CAM had high expression found in 62.30% (n = 38) of the cranial metastatic tumors, and low expression in 37.70% (n = 23) tumors. L1CAM expression was significantly correlated with the brain metastasis number (p = 0.028) and Lung-molGPA score (p = 0.042). Other clinicopathological factors, including gender, age, smoking history, extracranial transfer, metastatic brain location, KPS score, systemic therapy and radiotherapy before resection, and gene alterations in brain metastases, did not significantly affect the L1CAM expression (p > 0.05).

**Table 1:** L1CAM expression and clinicopathological parameters in brain metastases from lung adenocarcinoma.

Variables	Case, n°	L1CAM expression		χ2	p-value
		High (n = 38)	Low (n = 23)		
**Gender**					
Male	35	25	10	2.916	0.088
Female	26	13	13		
**Age (years)**					
≥ 70	7	5	2	0.013	0.908
< 70	54	33	21		
**Smoking history**					
No	25	13	12	1.912	0.167
Yes	36	25	11		
**KPS scores**					
≤ 70	9	7	2	0.443	0.506
> 70	52	31	21		
**BM locations**					
Supra.	42	23	19	3.258	0.071
Infra/Supra-Infra.	19	15	4		
**BM number**					
1-3	43	23	20	4.812	**0.028**
> 3	18	15	3		
**Extracranial transfer**					
No	54	32	22	0.892	0.345
Yes	7	6	1		
**Presurgical systemic therapy**^a^					
Yes	6	4	2	0.000	1.000
No	55	34	21		
**Presurgical radiotherapy**^b^					
Yes	8	5	3	0.000	1.000
No	53	33	20		
**Gene alterations in BM**					
EGFR mutation	25	13	12	2.187	0.335
ALK rearrangement	5	4	1		
Neg. or Unknow	31	21	10		
**Lung-molGPA**					
≤ 3	39	28	11	4.155	**0.042**
> 3	22	10	12		

L1CAM, L1 Cell Adhesion Molecule; KPS, Karnofsky Performance Status; BM, Brain Metastasis; Supra, Supratentorial; Infra/Supra-infra, Infratentorial or both Supratentorial and Infratentorial; Neg. or Unknow, Negative or Unknow; EGFR, Epidermal Growth Factor Receptor; ALK, Anaplastic Lymphoma Kinase; Lung-molGPA, the Updated Graded Prognostic Assessment for Lung Cancer Using Molecular Markers.

a Including chemotherapy, molecularly targeted therapy, and immunotherapy.

b Including stereotactic radiosurgery and whole brain radiation therapy.

### L1CAM expression predicting OS in brain metastases after resection

**Fig. 2:**
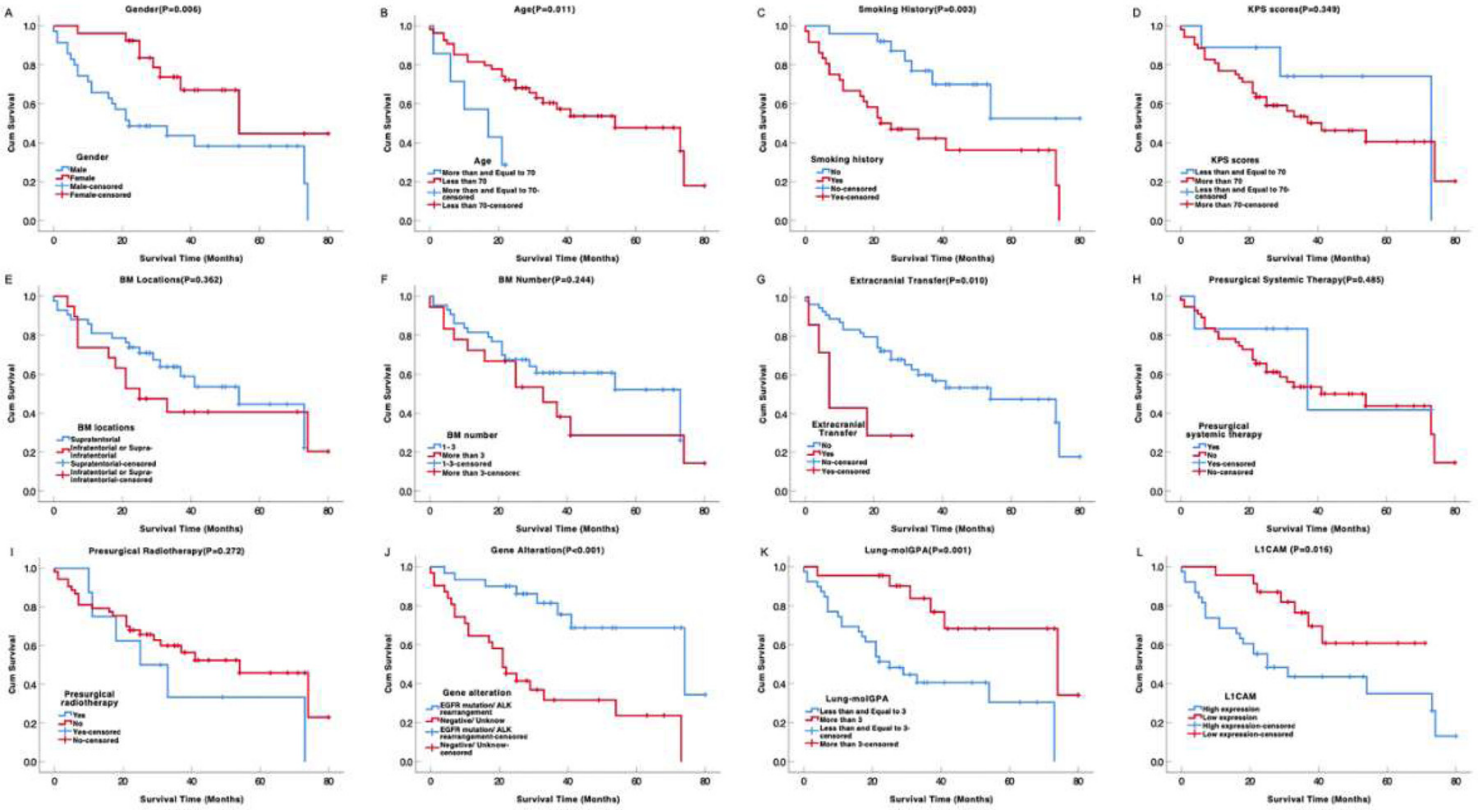
Kaplan-Meier curves showing survival time by clinic-pathological parameters for surgically resected brain metastases from lung adenocarcinoma. L1CAM high expression (L) was associated with unfavorable survival time. L1CAM, L1 Cell Adhesion Molecule.

In the total cases, the median OS for 61 patients with brain metastases from lung adenocarcinoma after neurosurgical resection is 41.000 months (SE = 13.019, 95% CI 15.483‒66.517). L1CAM high expression was associated with unfavorable OS (p = 0.016) in univariate analysis (Table 2). The median OS for brain metastasis with L1CAM high expression was only 25.000 months (SE = 7.159, 95% CI 10.967‒39.033) While the OS for L1CAM low expression did not reach the median because the death rate in this subgroup was lower than 50% (Mean OS: 54.857 months, SE = 4.964, 95% CI 45.128‒64.585 in L1CAM low expression subgroup). In addition, the univariate analysis also showed gender, age, smoking history, extracranial transfer, gene status, and Lung-molGPA were correlated with poor OS (Table 2 and Fig. 2). All other clinicopathological parameters were not associated with patient prognosis. Furthermore, the prognostic impact of L1CAM expression was independent of gender, age, smoking history, extracranial transfer, gene status, and Lung-molGPA in a multivariate Cox regression analysis for OS (Table 2). And multivariate analysis showed age and extracranial transfer were also the independent prognostic factors for brain metastasis with neurosurgical resection.

**Table 2:** Univariate and multivariate analyses of prognostic factors associated with OS in brain metastases after resection.

	Univariate analysis		Multivariate cox analysis			
Variables	χ2	p-value	B	p-value	Exp (B)	95% CI
**Gender** (Male, Female)	7.408	**0.006**	-0.616	0.551	0.540	0.071‒4.105
**Age** (≥ 70, < 70)	6.474	**0.011**	1.440	**0.009**	4.220	1.433‒12.424
**Smoking history** (No, Yes)	8.787	**0.003**	-1.591	0.129	0.204	0.026‒1.590
**KPS scores** (≤ 70, > 70)	0.878	0.349	‒	‒	‒	‒
**BM locations** (Supra. Infra/Supra-infra)	0.833	0.362	‒	‒	‒	‒
**BM number** (1‒3, > 3)	1.356	0.244	‒	‒	‒	‒
**Extracranial transfer** (No, Yes)	6.659	**0.010**	-1.254	**0.022**	0.285	0.098‒0.835
**Presurgical systemic therapy**^a^ (Yes, No)	0.488	0.485	‒	‒	‒	‒
**Presurgical radiotherapy**^b^ (Yes, No)	1.208	0.272	‒	‒	‒	‒
**EGFR/ALK gene status in BM** (Pos., Neg./unknown)	15.268	**< 0.001**	-1.213	0.127	0.297	0.063‒1.411
**Lung-molGPA** (≤ 3, > 3)	10.253	**0.001**	0.048	0.957	1.049	0.181‒6.085
**L1CAM expression** (High, Low)	5.757	**0.016**	0.961	**0.037**	2.641	1.059‒6.453

L1CAM, L1 Cell Adhesion Molecule; KPS, Karnofsky Performance Status; BM, Brain Metastasis; Supra, Supratentorial; Infra/Supra-infra, Infratentorial or both Supratentorial and Infratentorial; Neg. or Unknow, Negative or Unknow; EGFR, Epidermal Growth Factor Receptor; ALK, Anaplastic Lymphoma Kinase; Lung-molGPA, the Updated Graded Prognostic Assessment for Lung Cancer Using Molecular Markers; CI, Confidence Interval.

a Including chemotherapy, molecularly targeted therapy, and immunotherapy.

b Including stereotactic radiosurgery and whole brain radiation therapy.

## Discussion

In the clinic, neurosurgical resection is always indicated in the treatment for brain metastasis from lung cancer, especially for the symptomatic, large, or accessible solitary lesions or in the circumstances, there is a single large lesion that is life-threatening or produces mass effect among multiple lesions. Recently, the latest Lung-molGPA score incorporating gene alteration data (EGFR mutation/ALK rearrangement) is widely used to assess patient survival in NSCLC with brain metastases and facilitate clinical decision making.^14^ However, Lung-molGPA is not specifically designed for brain metastases with neurosurgical resection. Moreover, current research about the prognosis assessment of brain metastases mainly involves the clinic-pathological parameters of patients,^18^ cranial metastatic lesion-related molecular markers are not available and incorporated in the current prognosis system. A host of studies have shown that L1CAM is potentially involved in unfavorable survival in human malignancies of several different samples.^4^ Thus, in the present study, the authors firstly investigated the relationship between L1CAM expression in cranial metastatic lesions and clinicopathological parameters in brain metastases after neurosurgical resection and further tested the hypothesis that L1CAM expression was relevant for survival time in this subtype of patients.

In the present case series, L1CAM high expression was found in 62.30% of the cranial metastatic tumors from lung adenocarcinoma, with low expression in 37.70% of tumors. To the best of our knowledge, it is the first report that investigates the L1CAM expression in cranial metastatic tumors from lung cancer. The L1CAM expression in the primary lung lesion of each patient was not concurrently examined in this study because of the absence of lung adenocarcinoma samples, which is a limitation of the present study. Verena Tischler and his colleagues had indicated that 25% of NSCLC showed positive L1CAM expression, and the percentage of positive L1CAM expression could rise to 40% when tumors developed into pM1 category, indicating metastases occurred.^5^ Another team also found that 34.9% of NSCLC patients had L1CAM-positive expression and more than half of M1 stage tumor showed L1CAM-positive expression.^19^ It is unclear the exact molecular mechanisms leading to the L1CAM up-regulation in brain metastasis or primary NSCLC. Previous studies in multiple human cancers and cell lines have described several potential pathways, including Wnt/beta-catenin-TCF signaling,^4^^,^^20^ P53/Slug signaling,^21^ AXL-ABL2-TAZ feed-forward axis^22^ et al. More research has been designed in the present study's laboratory in order to reveal the underlying mechanism of brain metastasis.

Previous studies about prognostic factors in brain metastases mainly include clinic-pathological variables such as patient age, extracranial metastases, EGFR/ALK alterations and Lung-molGPA score,^14^ which have also been further demonstrated by the present study. Moreover, in the present study, the authors provide evidence that L1CAM expression is significantly correlated with prognosis in brain metastasis after surgical resection. L1CAM is an independent predictor of survival for brain metastases after neurosurgical resection. Patients with L1CAM high expression had unfavorable OS in comparison with the one with L1CAM low expression. These data are in line with previously published prognostic data on L1CAM expression in other human malignancies, such as NSCLC,^5^ gastric cancer,^10^ colorectal cancer,^4^^,^^23^ endometrial carcinoma,^9^ and glioblastoma.^7^^,^^13^ In addition, the Human Protein Atlas database (https://www.proteinatlas.org/) has also indicated that L1CAM is a prognostic marker.

The present study has also shown L1CAM expression is relevant to the number of brain metastases and Lung-molGPA score. Actually, to some extent, either the number of brain metastases or Lung-molGPA score reflects the severity and invasion of the tumor. For example, it has been reported by others that the patients with good Lung-molGPA (score > 3) achieved a survival benefit.^14^ Furthermore, recent reports have indicated that L1CAM plays an important role in the severity and invasion of the metastatic tumor by facilitating vascular co-option, promoting migration, outgrowth and colonization of tumor cells in the brain parenchyma.^24^^,^^25^ The therapy targeting the regulation of L1CAM expression may have important clinical implications and has been investigated *in vivo* and *in vitro* trials.

In conclusion, the authors demonstrate that a subset of brain metastases from lung adenocarcinoma aberrantly expresses L1CAM. L1CAM is a novel independent prognostic factor for brain metastasis from lung adenocarcinoma after neurosurgical resection.

## Authors' contributions

Jia-Wei Wang: Research design, data collection and paper writing; Song-Quan Wang, Zhuo-Yi Wu, Qi Liu: Data analysis; Qing Yuan, Hong-Qing Cai, Jing-Hai Wan: Research design, pathological analysis and data collection.

## Ethics committee name and study protocol number

Ethics Committee of the Cancer Hospital, Chinese Academy of Medical Sciences (Reference number NCC2021C-516/22/052-3253).

## Conflicts of interest

This work was supported by grants from Beijing Xisike Clinical Oncology Research Foundation (Y-QL202101-0094), National Natural Science Foundation of China (82072803) and CAMS Innovation Fund for Medical Sciences (2021-I2M-1-012).
